# Phase change material-based tunable Fano resonant optical coatings and their applications

**DOI:** 10.1515/nanoph-2023-0723

**Published:** 2024-01-17

**Authors:** Kandammathe Valiyaveedu Sreekanth, Sambhu Jana, Mohamed ElKabbash, Ranjan Singh, Jinghua Teng

**Affiliations:** Institute of Materials Research and Engineering (IMRE), Agency for Science, Technology, and Research (A*STAR), 2 Fusionopolis Way, Singapore 138634, Republic of Singapore; Division of Physics and Applied Physics, School of Physical and Mathematical Sciences, Nanyang Technological University, 21 Nanyang Link, Singapore 637371, Republic of Singapore; Centre for Disruptive Photonic Technologies, The Photonic Institute, 50 Nanyang Avenue, Singapore 639798, Republic of Singapore; Wyant College of Optical Sciences, University of Arizona, Tucson, AZ, 85721, USA

**Keywords:** thin film coatings, phase change materials, Fano resonance, structural coloring, hybrid thermal-electric power generation

## Abstract

Thin-film coatings offer a scalable optical platform, as compared to nanopatterned films, for various applications including structural coloring, photovoltaics, and sensing. Recently, Fano resonant optical coatings (FROCs) have gained attention. FROCs consist of coupled thin film nanocavities composed of a broadband and a narrowband optical absorber. The optical properties of FROCs can be dynamically adjusted using chalcogenide phase change materials (PCM). Switching the structural states of PCM layers in the cavity between amorphous and crystalline states, the Fano resonance supported by FROC can be modulated in terms of resonance wavelength, intensity, and bandwidth. This review discusses the scientific and technological facets of both passive and active FROCs for applications in structural coloring and spectrum-splitting filters. We explore electrically tunable FROCs for dynamic color generation and optical steganography. Furthermore, we discuss the utilization of passive and active FROCs as spectrum-splitting filters to mitigate the drop in photovoltaic efficiency of solar cells due to heating and for hybrid thermal-electric power generation.

## 1 Introduction

Chalcogenide phase change materials (PCMs) can undergo a phase transition in response to external conditions such as temperature and are highly promising in active and reconfigurable nanophotonic devices due to their large optical property changes during the structural transition between amorphous (Amp) and crystalline (Cry) [1]. The prototype PCM, Ge–Sb–Te (GST), and its family materials have attracted great attention because they offer energy-efficient and non-volatile tuning across a broad frequency band from ultraviolet (UV) to terahertz (THz) upon stimulation by optical and electrical pulses [2]–[11]. The unique features of PCMs are fast-switching speed, a highly stable switched state, and low loss at infrared (IR) to THz spectral band. These distinguished characteristics combined with compatibility with the complementary metal-oxide semiconductor (CMOS) process make PCMs suitable for a wide range of reconfigurable nanophotonics applications including memory devices, spatial light modulators, and flat lenses. The extensive research in recent years can be categorized into two main domains: (i) the material aspects of PCMs, where significant advancements have been made in understanding material properties and structural transformations during phase transition and (ii) various device configurations to enhance light–matter interactions and to achieve tunability across different platforms such as metasurfaces and on-chip photonic circuits [11]. Simultaneously, ongoing efforts involve optimizing these devices with the help of emerging technologies like machine learning to design and develop electrically reconfigurable flat optics components [12]. The future direction of PCM research would be on advancing energy efficiency, switching speed, and endurance within these devices [11].

The thin film optical coatings based on metallic films, anti-reflective coatings, transmission filters, light absorbers, and dielectric mirrors have recently attracted much interest from the scientific community since they provide a lithography-free method to develop cost-effective and scalable photonic devices [13]–[16]. In most of the metal-dielectric cavities consisting of asymmetric Fabry−Perot (FP) cavities, perfect absorption takes place when the light is critically coupled to the cavity. Since the transmission is zero in metal-dielectric cavities, the critical light coupling can be realized by suppressing reflection via thin film interference. By using this feature, thin film cavities have been widely employed for reflective structural coloring [16]–[18], hydrogen sensing [19], [20], and enhancing the light–matter interaction in atomically thin two-dimensional (2D) materials [21]–[23]. In addition, the singular phase realized at the critical coupling angle has been utilized for ultra-sensitive bio/chemical sensing applications [24]–[26]. Moreover, the optical properties of metal-dielectric cavities can be dynamically tuned by replacing the dielectric layers with active materials such as PCMs [27]–[33]. In this direction, various PCM-integrated thin film coatings have been developed for tunable structural coloring [27]–[31] and sensing applications [32], [33].

Recently, a new type of thin film optical coating known as Fano resonant optical coating (FROC) was developed by ElKabbash et al. [34]. The unique and distinguishing optical properties of FROCs cannot be reproduced with conventional thin film coatings. Fano resonance results from the resonant destructive interference between a narrowband resonance and the continuum. FROCs are produced by coupling a broadband nanocavity which represents the continuum, with a narrowband Fabry–Perot nanocavity which represents the discrete state. The resonant interference between the nanocavities produces a Fano resonance. One of the unique properties of FROCs is that they act as a filter that reflects and transmits the same color, i.e., they act as a beam-splitting color filter [34]–[40]. Opaque and highly reflective FROCs represent state-of-the-art structural coloring as they produce colors spanning a wide color gamut with high brightness, high purity, and controlled iridescence [36]. In the context of clean energy applications, FROCs were used as a spectrum splitting filter in hybrid thermal-electric solar power generation to divide the solar spectrum into a photovoltaic band and a thermal band and thus to improve the efficiency of power generation and photovoltaic cell lifetime [34]. Moreover, FROCs were proposed for polarization detection [41] and as a potential surface-enhanced Raman spectroscopy (SERS) substrate for biosensing applications [42].

Here, we review the progress in dynamically tuning the optical properties of FROCs by incorporating PCMs. We investigate the tuning of the resonance wavelength, linewidth, and intensity of the Fano resonance in reflective and transmissive FROCs, and in particular, the electrically tunable PCM-based FROCs and their applications in dynamic structural coloring and optical steganography. Moreover, we discuss the use of FROC to develop spectrum-splitting filters to mitigate the drop in photovoltaic efficiency of solar cells due to heating and for hybrid thermal-electric power generation.

## 2 FROC and PCM-based tunable FROC

**Figure 1:**
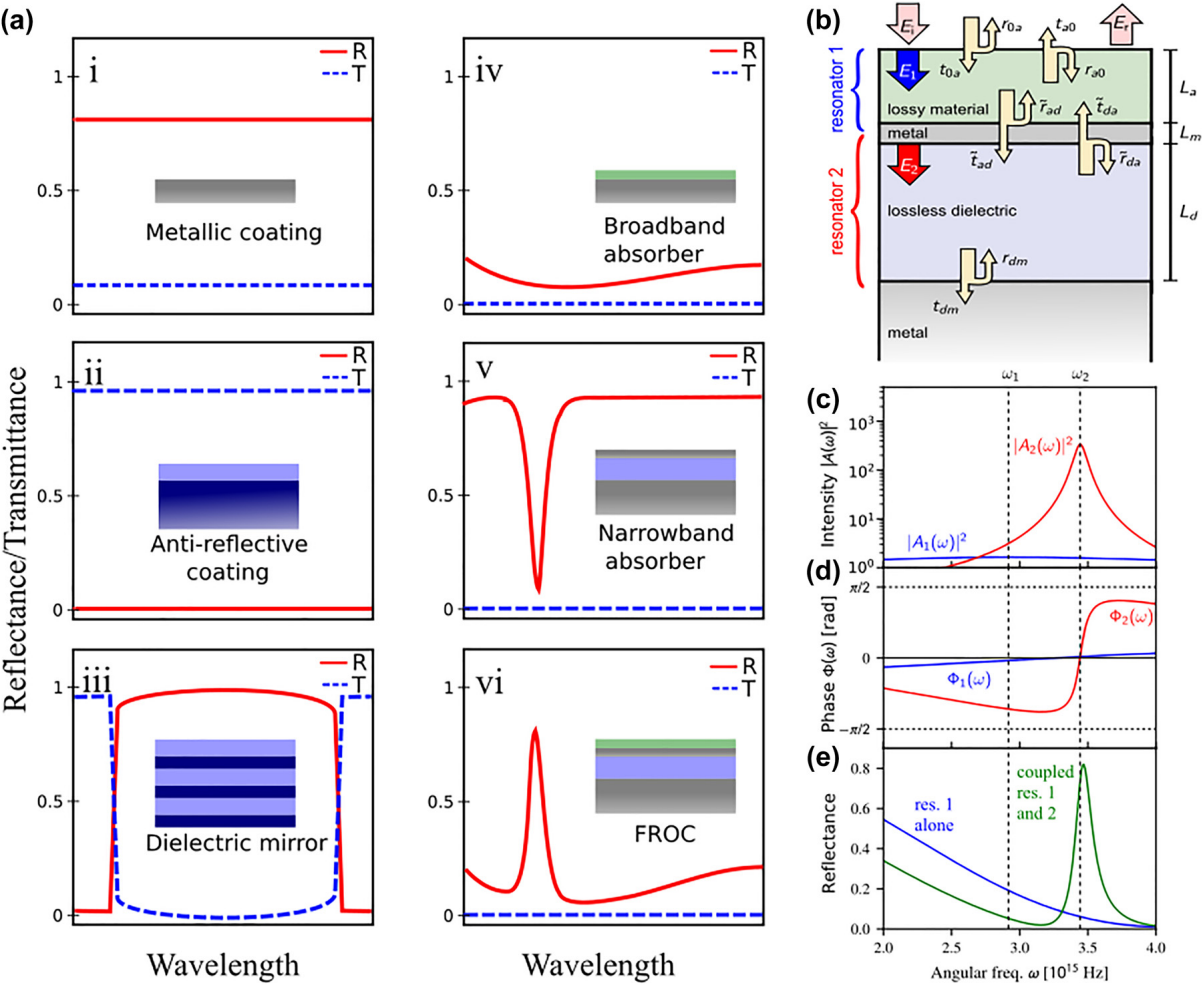
Demonstration of Fano resonance in thin film coatings. (a) Schematics of the reflectance (*R*) and transmittance (*T*) of the conventional thin-film coatings and FROC. (b) A schematic of the FROC geometry made up of two weakly coupled resonators with resonator 1 representing a broadband absorber and resonator 2 representing a narrowband absorber. (c) Calculated oscillator intensities, (d) Corresponding oscillator phases, and (e) Reflectance of the two coupled resonators. Adapted with permission from ElKabbash et al. [34]. Copyright 2021, Nature Springer.

In contrast to conventional thin-film coatings (as shown in Figure 1a(i)–(v)), a FROC structure (Figure 1a(vi)) can excite Fano resonance. The Fano resonance is realized by coupling a broadband (Figure 1a(iv)) and a narrowband (Figure 1a(v)) absorber, representing the continuum and discrete states, respectively. The coupled oscillator theory can explain the observed Fano resonance (Figure 1b), as the FROC comprises two weakly coupled resonators with resonator 1 and 2 representing the broadband and narrowband absorber, respectively. The metal layer between the lossy material and lossless dielectric determines the coupling strength. As shown in Figure 1d, the Fano resonance is realized due to the destructive interference between the constant and steep varying phases of the overlapping broadband and the narrowband absorbers, which leads to an asymmetric Fano line shape. Interestingly, the reflection and transmission spectra of FROC are the same with resonance wavelength overlaps, which is also different from conventional thin-film coatings [34]. Moreover, FROC can be made iridescence and iridescence-free thin film coating by selecting the lossless dielectric as low- and high-refractive-index material, respectively. Thus, the low-index FROC-based iridescent structural color is useful for the anti-counterfeiting measures used in many currencies and high-index FROC-based iridescent-free structural color can be used for the other structural coloring applications.

**Figure 2:**
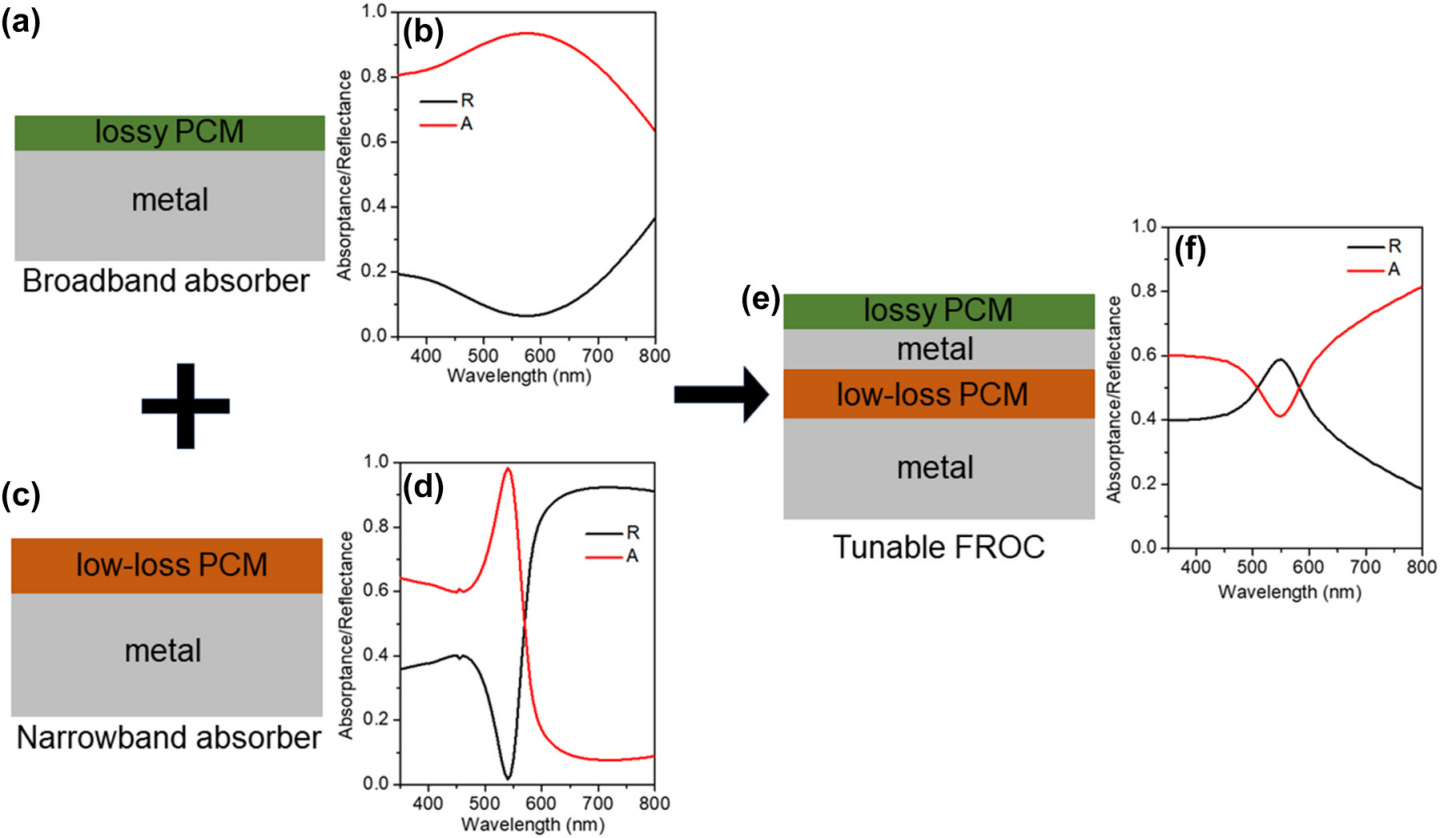
Demonstration of tunable Fano resonance using PCMs. (a) Schematic of a PCM-based broadband absorber and (b) its absorption and reflection spectra. (c) Schematic of a PCM-based narrowband absorber and (d) its absorption and reflection spectra. (e) Schematic of a PCM-based FROC and (f) its absorption and reflection spectra. Adapted with permission from Sreekanth et al. [37]. Copyright 2021, American Chemical Society.

A tunable FROC can be realized by integrating PCM into the broadband and narrowband absorbers [37]. As shown in Figure 2a, the tunable broadband absorber consists of a thin layer of lossy PCM on a metal, which gives broadband absorption in the visible wavelength (Figure 2b), while the tunable narrowband absorber consists of a thin layer of ultra low-loss PCM on a metal (Figure 2c), corresponding to narrowband absorption spectrum shown in Figure 2d. The tunable FROC is then developed by placing the broadband absorber on the narrowband absorber (Figure 2e). It is important to note that the absorption and reflection spectra of FROC is opposite to that of broadband and narrowband absorbers, as shown in Figure 2f.

**Figure 3:**
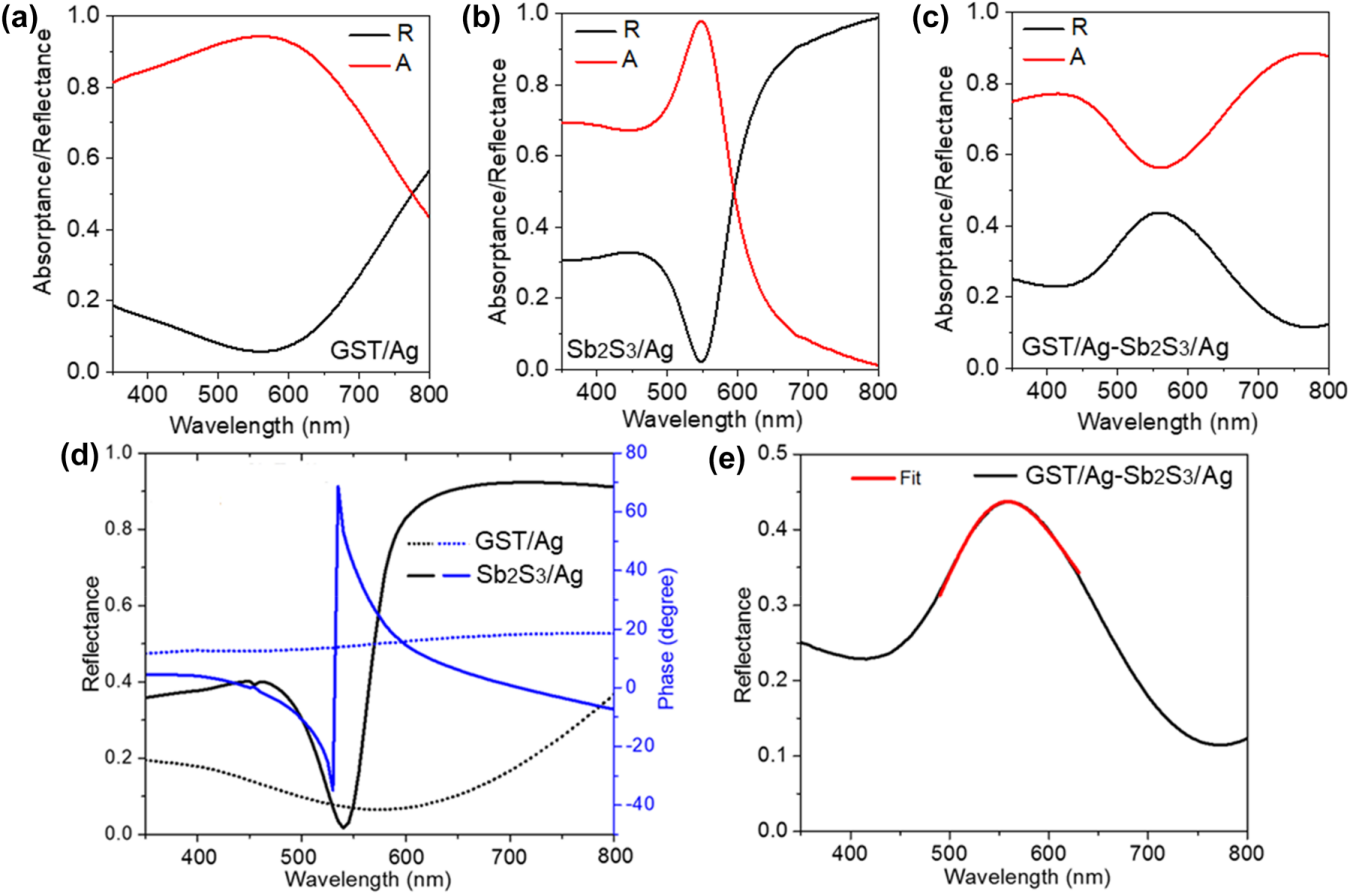
Measured absorption and reflection spectra of (a) GST/Ag broadband absorber, (b) Sb_2_S_3_/Ag narrowband absorber, and (c) GST/Ag–Sb_2_S_3_/Ag FROC. (d) Calculated reflection and phase spectra of GST/Ag broadband and Sb_2_S_3_/Ag narrowband absorbers. (e) The measured reflection spectrum of a GST (20 nm)/Ag (20 nm)-Sb_2_S_3_ (30 nm)/Ag (100 nm) FROC and fitting using an asymmetric Fano function. Adapted with permission from Sreekanth et al. [37]. Copyright 2021, American Chemical Society.

For experimental validation, Ge_2_Sb_2_Te_5_ (GST) and Sb_2_S_3_ thin films were used as the lossy and ultra low-loss PCM, respectively. With the phase transition from Amp to Cry, GST provides a huge extinction coefficient (*k*) variation [3] and Sb_2_S_3_ provides a large refractive index (*n*) variation (with low *k*) in the visible wavelengths [43]. The tunable broadband and narrowband absorbers were fabricated by depositing a thin GST layer (thickness ∼20 nm) on a ∼100 nm thick Ag and a ∼90 nm thick Sb_2_S_3_ film on a ∼100 nm thick Ag, respectively. The measured normal incidence broadband and narrowband absorption spectra of GST/Ag and Sb_2_S_3_/Ag are shown in Figure 3a and b, respectively. Figure 3c shows the reflection and absorption spectra of the combined geometry of GST/Ag–Sb_2_S_3_/Ag FROC at normal incidence, where the deposited thickness of GST, top Ag, Sb_2_S_3,_ and bottom Ag is ∼20 nm, ∼20 nm, ∼30 nm, and ∼100 nm, respectively. As shown in Figure 3e, the Fano line shape can be fitted with a Fano fitting formula, $\sigma \left(E\right)=A+D^{2}\frac{{\left(q+\Omega \left(E\right)\right)}^{2}}{1+\Omega^{2}\left(E\right)}$ , where *A* and *D* are the fitting parameters, *E* is the energy, *q* is the Fano parameter, and $\Omega \left(E\right)=2\left(E-E_{0}\right)/\Gamma$ with *E*_0_ and Γ are the resonance energy and width, respectively. Also, note that the PCM-based tunable FROC is an iridescence-free thin film coating as the refractive index of Sb_2_S_3_ is high [37].

## 3 FROC for reflective and transmissive structural coloring

**Figure 4:**
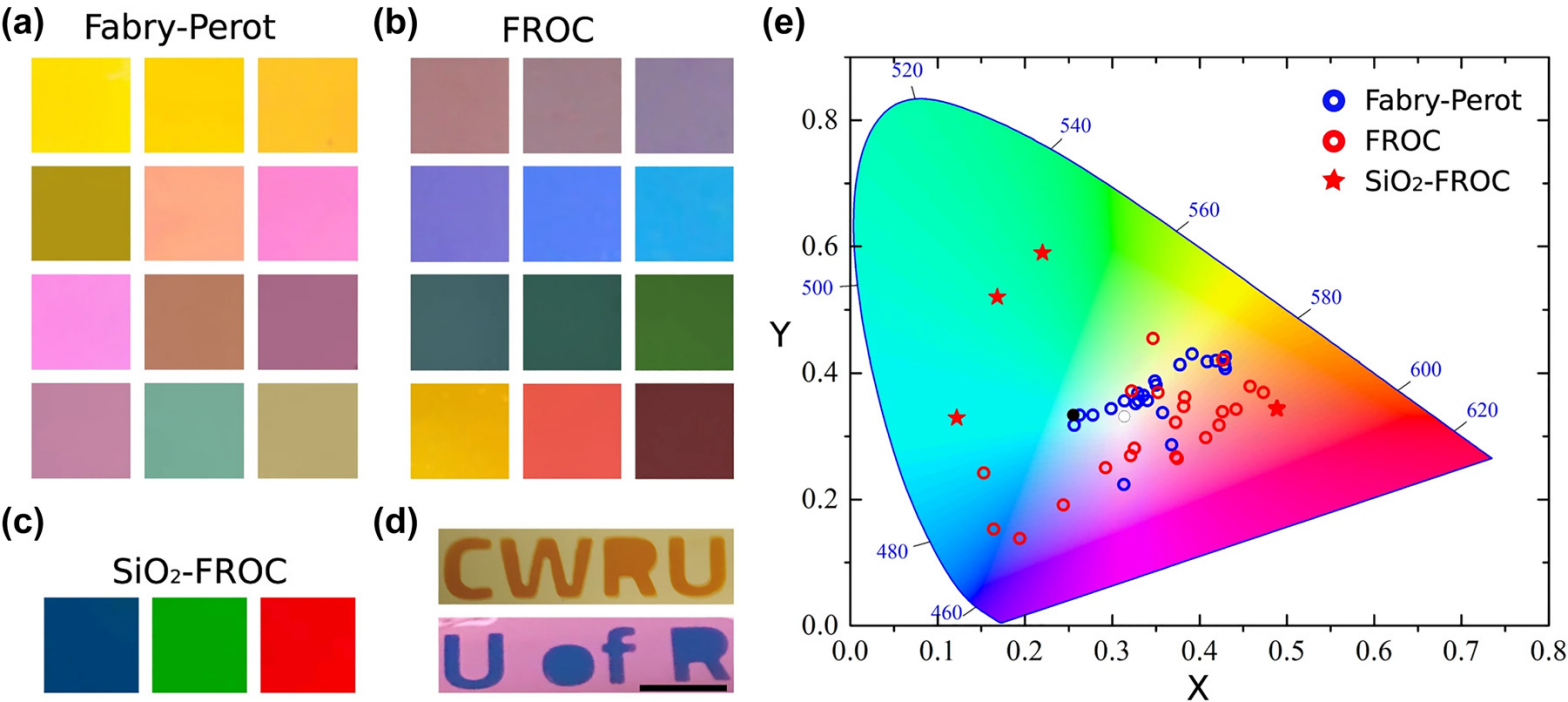
The colors for different thicknesses (35 nm–150 nm) of the TiO_2_ cavity layer of (a) F–P cavity, (b) FROC, and (c) capped SiO_2_ FROC for three colors. (d) Demonstration of color change by converting F–P cavity to FROC. (e) The CIE 1931 color space shows the colors corresponding to the reflection spectra of the F–P cavity and FROC for different thicknesses of the TiO_2_ cavity layer. Adapted with permission from ElKabbash et al. [36]. Copyright 2023, Nature Springer.

It has been reported that reflective structural coloring with iridescence-free and high-purity colors is possible with FROC due to its selective reflection [34]–[40]. In contrast to F–P cavity with different cavity layer (TiO_2_) thicknesses (Figure 4a), FROC can efficiently reflect blue, green, and red colors by changing the thickness of TiO_2_ cavity layer (Figure 4b). The color purity can be further enhanced by capping SiO_2_ (Figure 4c). As shown in Figure 4d, the color of F–P cavityis significantly changed by depositing a thin layer of lossy dielectric (15 nm Ge) on it, where the printed letters represent the FROC. Figure 4e shows the reflectance of the F–P cavity (blue dots) and FROC (red dots) for different thicknesses of the TiO_2_ cavity layer presented in the CIE 1931 color space. In short, FROC can produce high-purity blue, green, and red colors.

**Figure 5:**
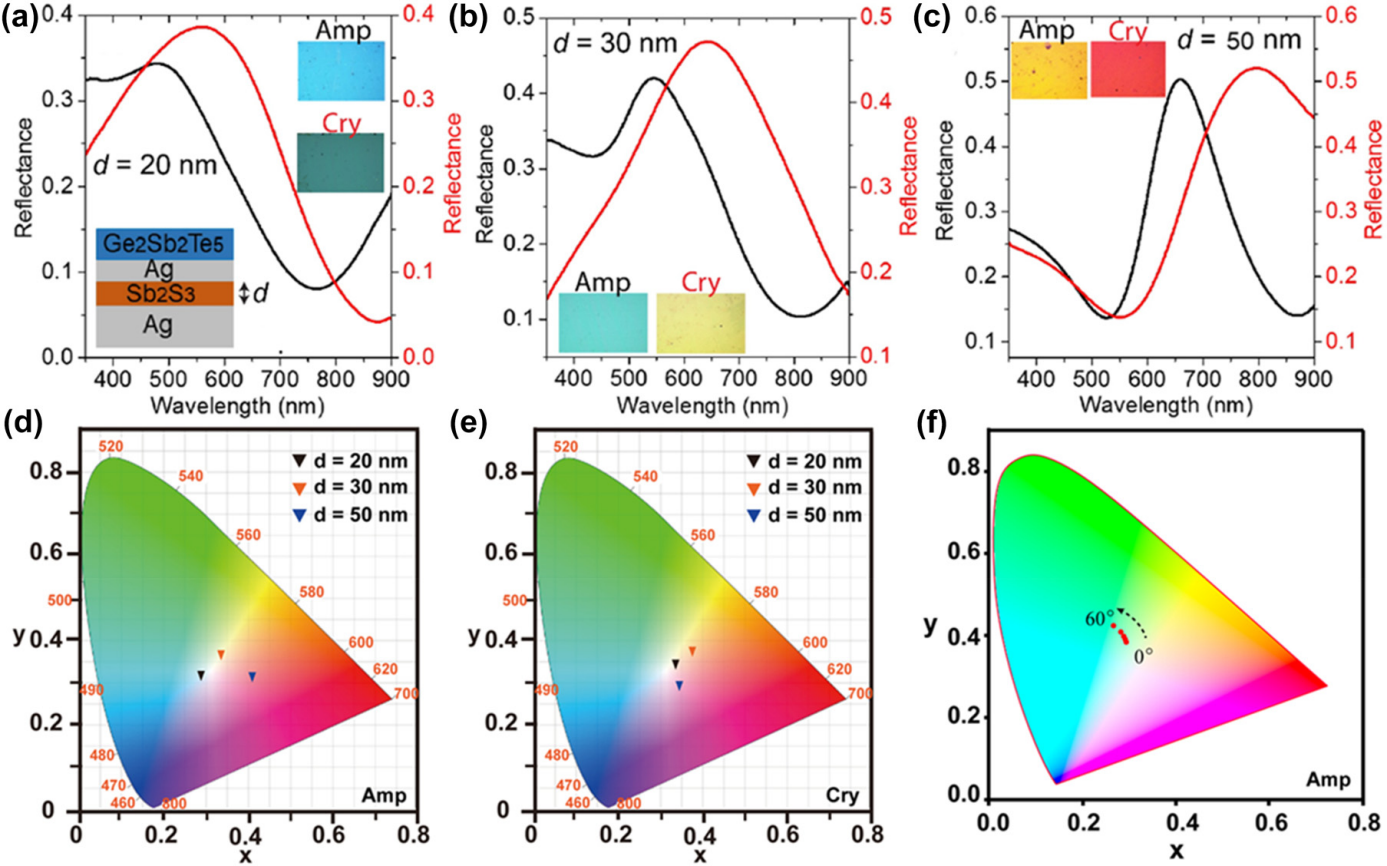
Tuning the Fano resonance of FROC by direct annealing, for different thicknesses of Sb_2_S_3_ cavity layer (a) 20 nm, (b) 30 nm, and (c) 50 nm. Optical microscope image of the corresponding color change obtained for Amp and Cry FROC is shown in the inset of the figures. The CIE 1931 color space shows the colors corresponding to the measured reflection spectra for different thicknesses of the Sb_2_S_3_ cavity layer for (d) Amp and (e) Cry FROC. (f) Color space for calculated angular reflection spectra of Amp FROC with Sb_2_S_3_ cavity layer thickness of 40 nm (0°–60°). Adapted with permission from Sreekanth et al. [37]. Copyright 2021, American Chemical Society.

We further discuss the importance of PCM-based FROCs for realizing tunable reflective and transmissive structural colors. Since the proposed FROC consists of two PCMs with different crystallization temperatures (≥150° for GST and ≥280 °C for Sb_2_S_3_), the FROC samples were annealed at a moderate temperature of 220 °C for the phase change of PCM layers, which indicates that Sb_2_S_3_ layer is partially crystallized. The reflective color tunability of FROC is investigated by slightly changing the thickness of Sb_2_S_3_ cavity layer of the FROC. In Figure 5a–c, we show the tunable reflective colors obtained with the phase transition of PCM layers for different thicknesses of the Sb_2_S_3_ layer. The peak wavelength of Fano resonance is red-shifted by slightly increasing the thickness of the Sb_2_S_3_ layer (*d* = 20–50 nm). As a result, wide colors ranging from blue to red are realized by switching the structural state of PCM layers from Amp to Cry. Also, note that the red-shifted wavelength increases with an increase in Sb_2_S_3_ layer thickness.

The measured reflectance spectra of the tunable FROC with different thicknesses (20 nm, 30 nm, and 50 nm) of the Sb_2_S_3_ cavity layer are presented in the CIE 1931 color space for the Amp (Figure 5d) and Cry (Figure 5e) phases. It is visible that the color change is obtained with the phase change of both PCM layers for three thicknesses of the Sb_2_S_3_ cavity layer. It is also clear that the color tunability increased with increasing Sb_2_S_3_ cavity thickness. In Figure 5f, we show the calculated angular reflection spectra in the color space for Amp FROC with a 40 nm thick Sb_2_S_3_ cavity layer. It can be seen that the structural color remains the same up to 40° and then changes slightly with a further increase of incidence angle from 50° to 60°. This proves that the PCM-based FROC is an ideal platform for obtaining high-purity iridescence-free structural colors. Moreover, the color purity can be increased and the oxidation issue due to annealing can be mitigated by capping the PCM-based FROCs with a thin layer of SiO_2_.

**Figure 6:**
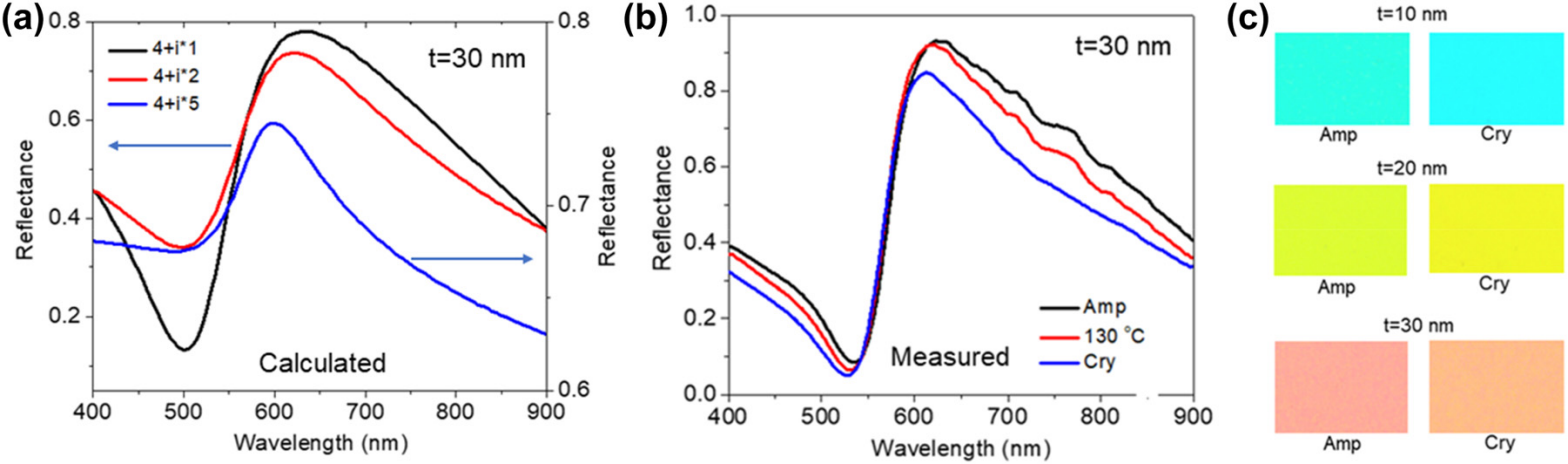
Linewidth narrowing using lossy PCM. (a) Calculated linewidth narrowing of Fano resonance with an increase in extinction coefficient (*k*) of lossy PCM. Here, ‘*t*’ represents the thickness of lossy PCM. (b) Measured linewidth narrowing of Fano resonance with phase transition of GST layer from Amp to Cry. (c) The observed high purity color with linewidth narrowing of Fano resonance for GST layer thickness, *t* = 10 nm, 20 nm, and 30 nm. Adapted with permission from Prabhathan et al. [38]. Copyright 2023, American Chemical Society.

As FROC produces colors through selective reflection, the generation of high-purity colors is possible by narrowing the linewidth of the Fano resonance. The linewidth of the FROC can be controlled by adjusting the structural parameters of the FROC, however, this is a static approach. In such instances, the color generated by the FROC undergoes a notable shift in wavelength due to a large Fano resonance shift [34]. Alternatively, Fano resonance linewidth can be dynamically tuned without the drastic shift of resonance wavelength by controlling the loss in the lossy PCM [38]. In Figure 6a, we show the calculated results of linewidth narrowing of Fano resonance with the increase in the imaginary refractive index (*k*) of lossy dielectric, where the real refractive index is kept constant. The linewidth is reduced with increasing *k*, with calculated linewidths are 260 nm, 225 nm, and 124 nm for the *k* values of 1, 2, and 5, respectively. To experimentally prove this concept, GST (*t* = 30 nm)/Ag (10 nm)/Sb_2_S_3_ (40 nm)/Ag (100 nm) reflective FROC was used; where the *k* value of GST continuously increases with increasing the annealing temperature. In this case, the FROC was annealed up to the crystallization temperature (160 °C) of GST, so that the refractive index of Sb_2_S_3_ would not change. Figure 6b shows the measured reflection spectra of Amp and annealed (130 °C and 160 °C [Cry]) FROC samples. As expected, the linewidth of Fano resonance continuously decreased with an increase in temperature. For different thicknesses of the GST layer, the high purity color obtained due to the phase change of the GST from Amp to Cry is shown in Figure 6c. For example, the greenish–yellow color produced by Amp FROC for *t* = 20 nm is changed to pure yellow color after the phase change of GST.

**Figure 7:**
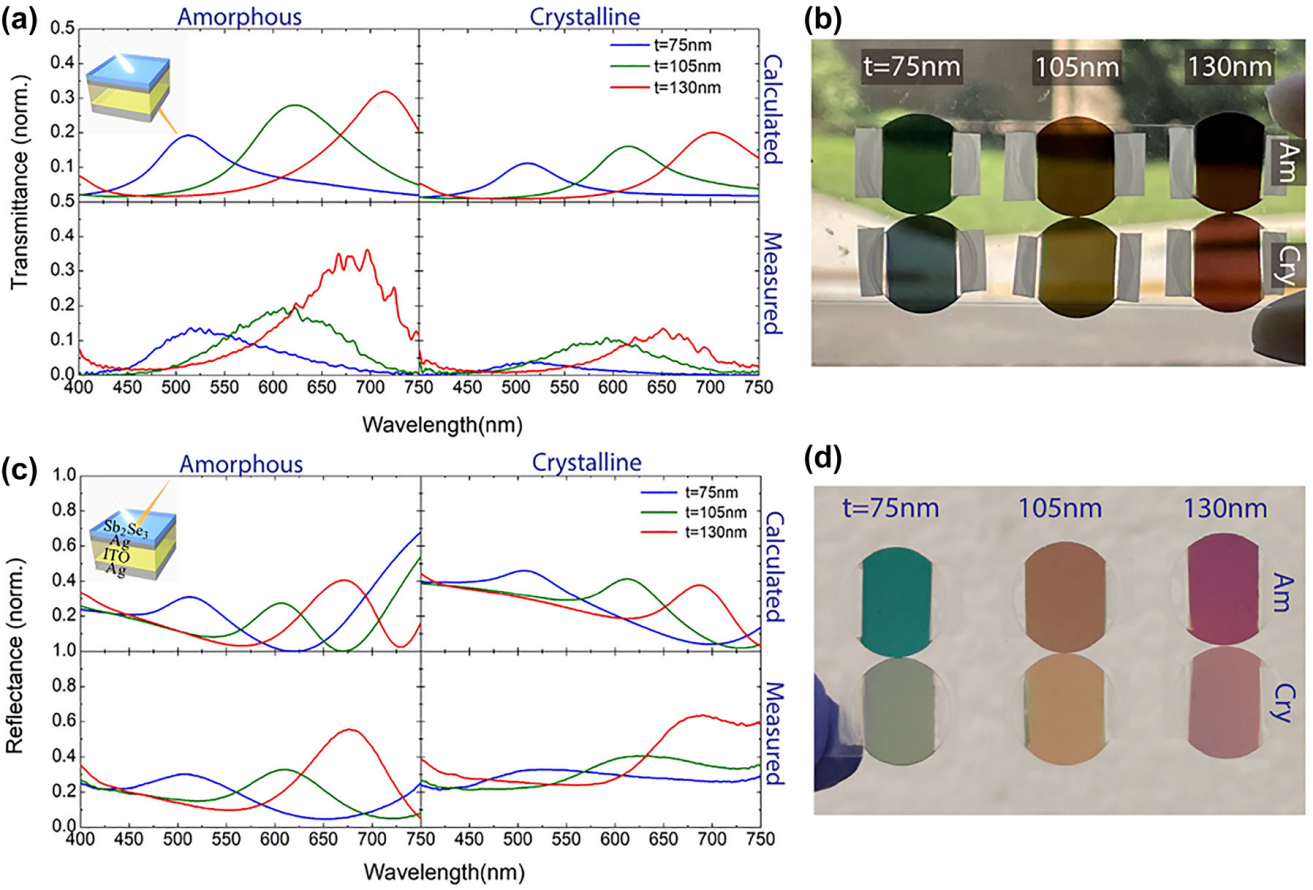
Tunable transmissive FROC using one PCM layer. (a) Calculated and measured transmission spectra of PCM-based transmissive FROC in Amp and Cry phases for different thicknesses of ITO cavity layer. (c) Calculated and measured reflection spectra of PCM-based reflective FROC in Amp and Cry phases for different thicknesses of ITO cavity layer. Schematic of the fabricated Sb_2_Se_3_/Ag/ITO/Ag FROC is shown in the inset of Figure (a) and (c). Picture of the (b) transmissive and (d) reflective PCM-based FROC samples for different thicknesses of the ITO cavity layer. Adapted with permission from Huang et al. [39]. Copyright 2023, Elsevier.

Recently, Huang et al. demonstrated tunable transmissive structural coloring using a PCM-based semi-transparent FROC [39]. To achieve sufficient transmitted intensity, Sb_2_Se_3_ PCM and ITO were used as the lossy dielectric layer and cavity layer, respectively. Note that the loss in both phases of Sb_2_Se_3_ is low in the visible wavelengths compared to GST. The developed tunable transmissive FROC is Sb_2_Se_3_ (17 nm)/Ag (30 nm)/ITO (*t*)/Ag (25 nm). Figure 7a shows the measured and calculated transmission spectra in both phases of Sb_2_Se_3_ for different thicknesses (*t* = 75 nm, 105 nm, and 130 nm) of the ITO cavity layer. The corresponding color in the Amp and Cry phase of Sb_2_Se_3_ is shown in Figure 7b. The reflective tunable colors of the Sb_2_Se_3_-based FROC for the same thicknesses of the ITO layer are also investigated (Figure 7c and d). As can be seen, the Fano resonance wavelength overlaps in transmission and reflection spectra; as a result, FROC shows almost the same color in transmission and reflection. However, the observed spectral shift due to the phase change of Sb_2_Se_3_ is very small compared to a dual PCM-based FROC. To obtain the large spectral tunability with a wide color change, the cavity layer of the FROC is better to be a PCM as well.

**Figure 8:**
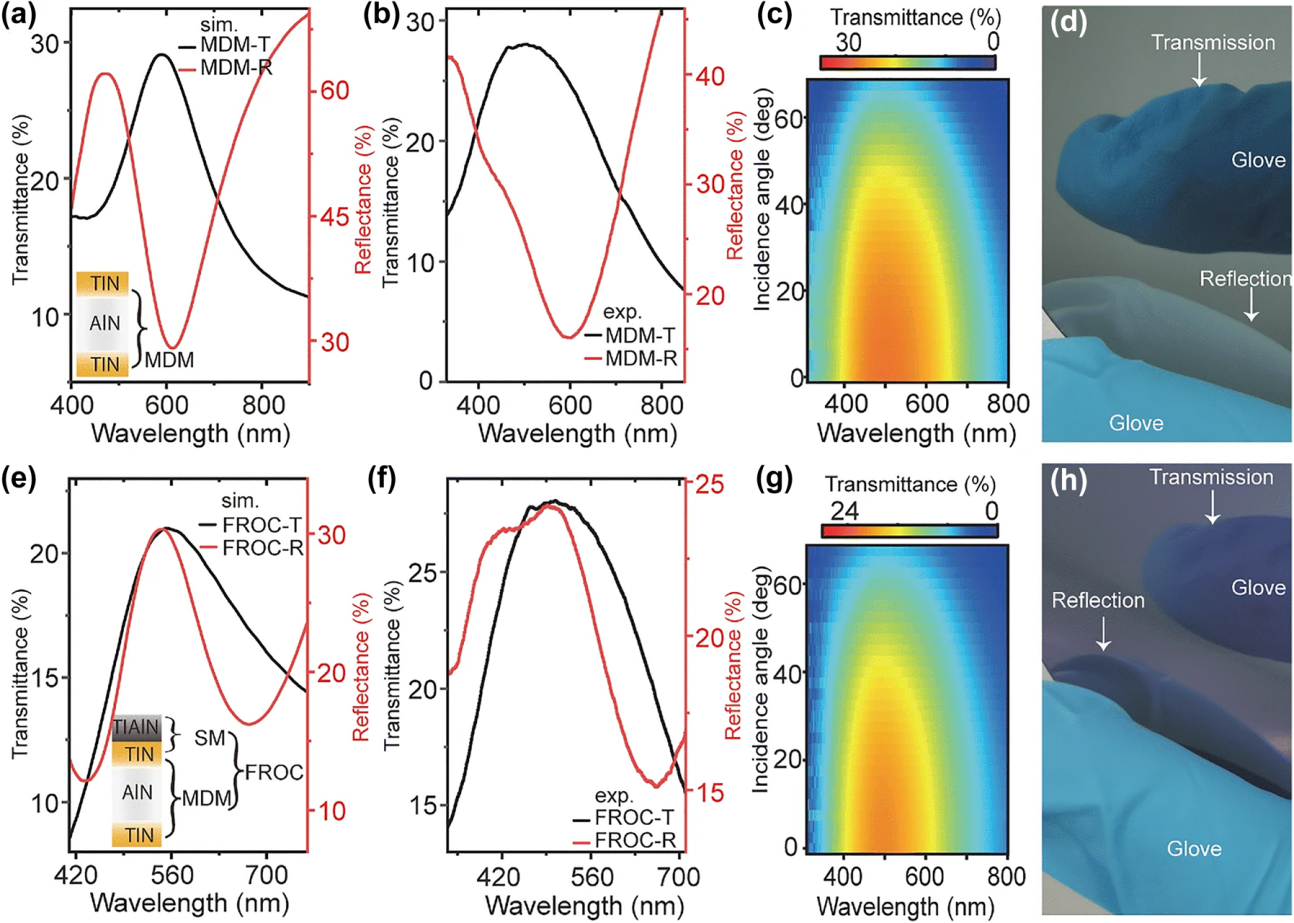
Transmittance and reflectance spectra of MDM film at normal incidence (a) calculated and (b) measured. Transmittance and reflectance spectra of FROC film at normal incidence (e) calculated and (f) measured. Measured angular transmittance of (c) MDM film and (g) FROC film. Photograph of reflected and transmitted color for (d) MDM film and (h) FROC. Adapted with permission from Geng et al. [40]. Copyright 2022, Springer.

Since the commonly used lossy dielectrics (Si, Ge, and GST) and noble metals (Au, Ag, and Al) are not robust enough to resist mechanical abrasion, wear-resistant thin film coatings are important for the practical applications of structural colors. Recently, a wear-resistant FROC structure based on extremely hard ceramic materials such as TiN, AlN, and TiAlN was proposed for structural coloring [40]. In this FROC, TiN, TiAlN, and AlN function as the reflecting metal layer, lossy dielectric layer and cavity layer, respectively. Figure 8 illustrates the colors produced in reflectance and transmittance using hard ceramic materials-based metal–dielectric–metal (MDM) and FROC coatings. In contrast to MDM film (Figure 8d), FROC produces the same color in reflection and transmission (Figure 8h). This wear-resistant FROC can also be made tunable by replacing the AIN cavity layer with Sb_2_S_3_.

## 4 Electrically tunable reflective structural coloring with FROC

**Figure 9:**
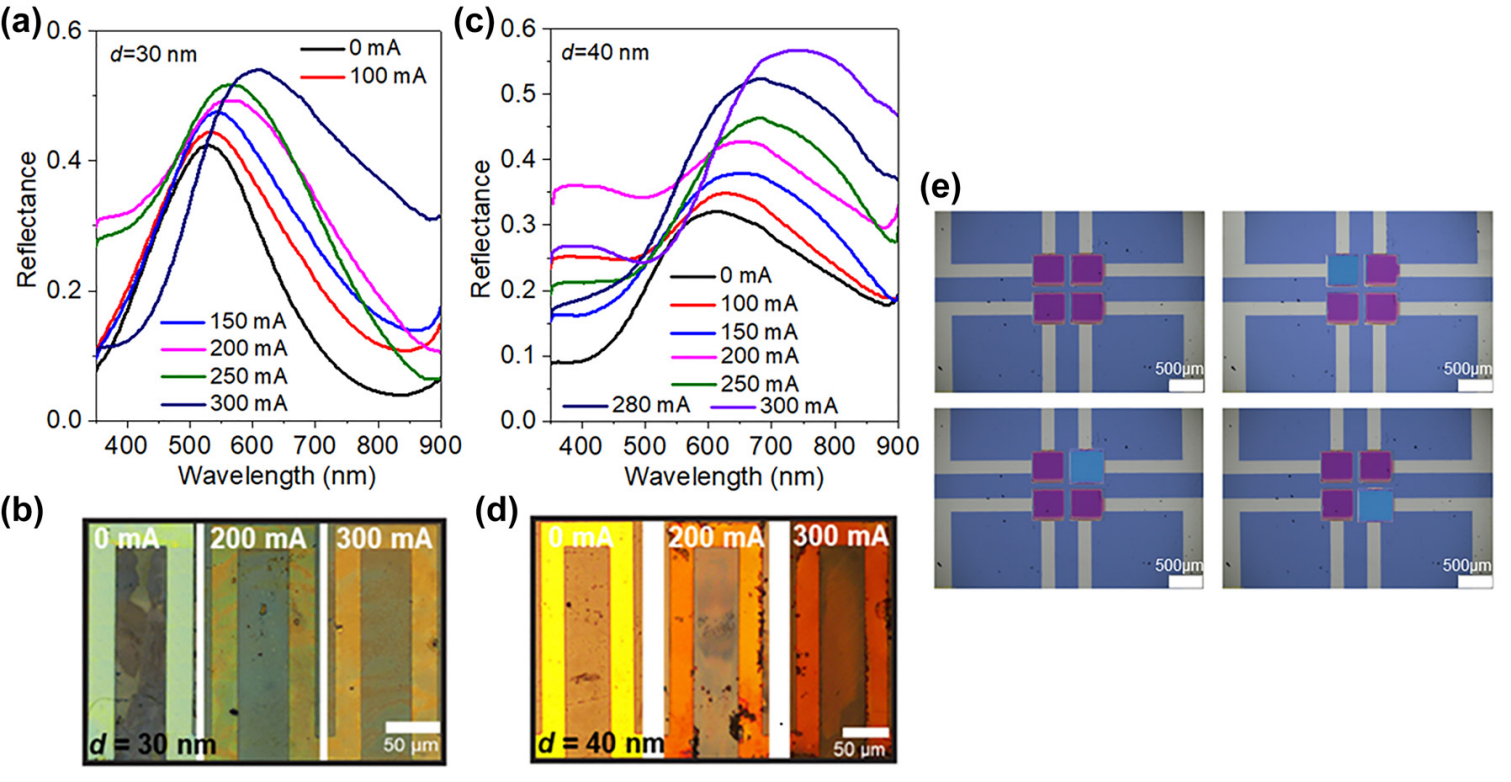
Electrical continuous tuning of Fano resonance of FROC with applied DC current for Sb_2_S_3_ cavity layer thickness of (a) 30 nm and (c) 40 nm. Optical microscopic image of the continuous color change obtained on FROC with increasing current (0–300 mA) for Sb_2_S_3_ cavity thickness of (b) 30 nm and (d) 40 nm. (e) Electrical individually addressable 2 × 2 pixel array of FROC with Sb_2_S_3_ cavity thickness of 15 nm. Adapted with permission from Sreekanth et al. [37] Copyright 2022, American Chemical Society and Prabhathan et al. [38]. Copyright 2023, American Chemical Society.

The electrically tunable reflective structural coloring was demonstrated by integrating the GST/Ag/Sb_2_S_3_/Ag FROC with a microheater [37], where the entire sample was annealed by electric current-induced heating via Joule heating mechanism (Figure 9). Here, the bottom metal layer itself is considered as the microheater, thus only the metallic bars of the microheater representing the FROC (Figure 9b–d). For the Sb_2_S_3_ cavity layer thickness of 30 nm and 40 nm, the electrically continuous forward (Amp to Cry) tuning of Fano resonance is shown in Figure 9a–c, respectively. With increasing current from 0 to 300 mA, a redshift of Fano resonance is obtained. Thus, the color change obtained for 30 nm and 40 nm is green to yellow (Figure 9b) and yellow to red (Figure 9d), respectively. The color tunability of the individually addressable 2 × 2 pixel array of FROC with a 15 nm thick Sb_2_S_3_ layer is shown in Figure 9e, where the violet color is changed to blue by applying a DC voltage of 10 V. In these studies, only electrical forward tuning of colors was demonstrated using DC current/voltage. To realize reconfigurable structural coloring devices, the FROC should be integrated with a precisely designed microheater, and high energy short electrical pulses are required [8], [44], [45].

## 5 Tunable FROC for optical steganography

Steganography is a technique where the messages are concealed in such a way that only the intended recipient can decode them [46]. In recent years, image-based optical steganography has received substantial attention, where a message is embedded in a digital image in a way that is not apparent to the naked eye [47]. The goal of image-based steganography is to generate a message that is hidden in an image in such a way that it cannot be detected by someone who is not aware of its presence within the image. In particular, multiplexing image-based optical steganography has been demonstrated using metasurfaces and plasmonic color reflectors [48], [49]. However, advanced nanofabrication techniques are required to develop these nano-structural coloring devices, which is not cost-effective and scalable. To solve this issue, the high purity and full gamut color generation ability of FROC can be used for optical steganography. Here, we discuss the use of PCM-incorporated tunable FROC as a potential active color reflector for optical steganography applications [38].

**Figure 10:**
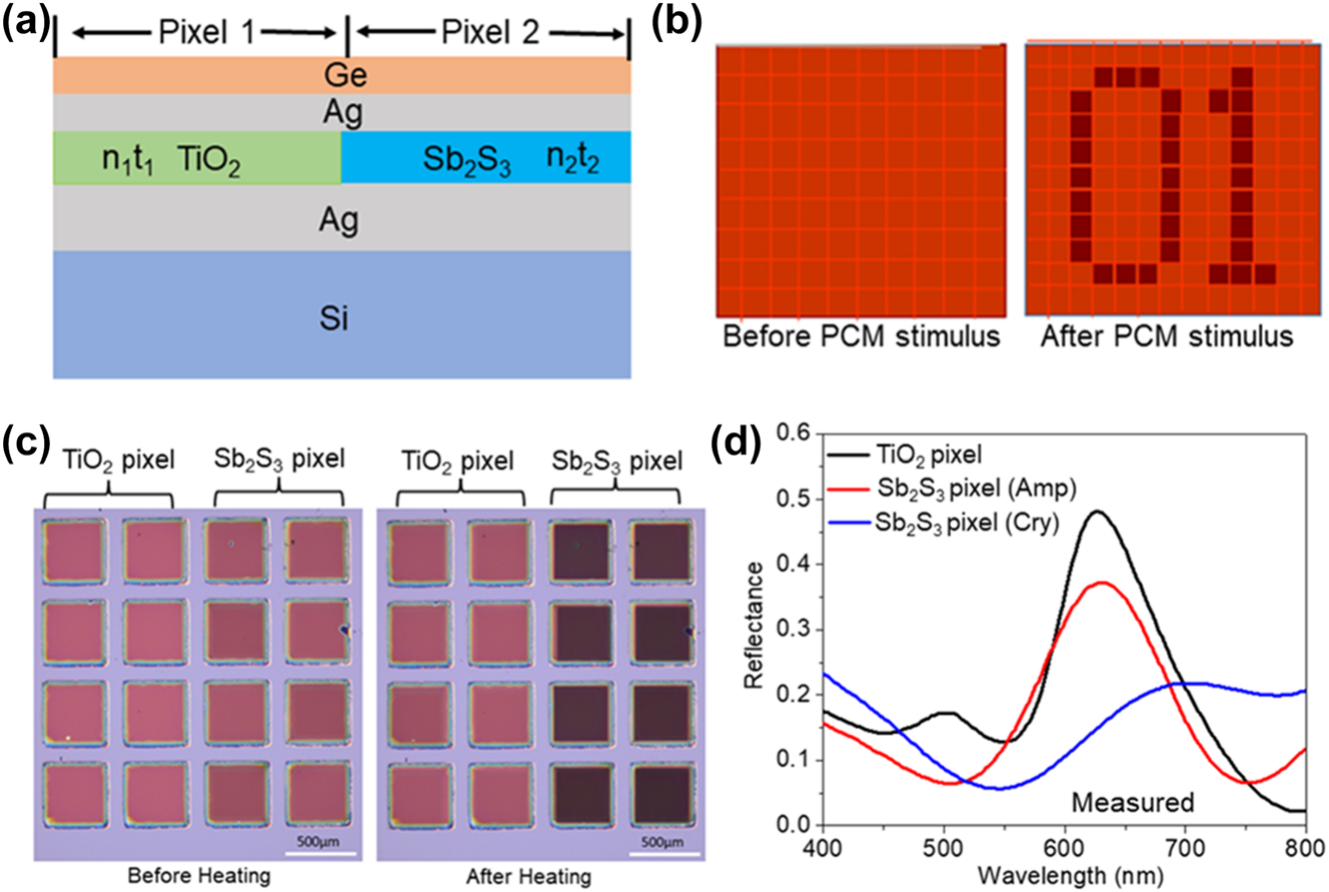
Demonstration of optical steganography using FROC. (a) Schematic of proposed FROC color pixels for a two-pixel image encryption. (b) A schematic of a steganographic reflector with multiple pixels before (left) and after (right) applying external stimulus. (c) Experimental demonstration of image encryption with FROC pixels before and after annealing. (d) The measured reflection spectrum of TiO_2_ pixel and Sb_2_S_3_ pixel in Amp and Cry phases. Adapted with permission from Prabhathan et al. [38]. Copyright 2023, American Chemical Society.

Figure 10a shows the schematic of fabricated FROC-based reflective color pixels for two-pixel image encryption. As can be seen, the cavity layer of Pixel 1 and Pixel 2 is made up of TiO_2_ and Sb_2_S_3_, respectively and the other layers of both pixels are the same with the deposited thickness of Ge, top Ag, and bottom Ag is ∼20 nm, ∼20 nm, and ∼100 nm, respectively. Here, Ge was selected as the lossy dielectric because the color change is only required with the phase change of the Sb_2_S_3_ cavity layer. For the Amp phase of Sb_2_S_3_, the thickness of TiO_2_ and Sb_2_S_3_ is selected in such a way that the optical path length condition matches (i.e., *n*_1_ *t*_1_ = *n*_2_ *t*_2_), as a result, the Fano resonance wavelength of both pixels match, and two pixels look uniform in color, which enables a camouflage property to it. With the applied external stimulus (temperature) on the FROC device, the color change only occurs in Pixel 2 due to the structural phase change of the Sb_2_S_3_ layer from Amp to Cry. Figure 10b further illustrates this approach, where the first picture shows the camouflaged image with uniform color for both pixels and the second picture shows the recovered image after the phase change of Sb_2_S_3_ layer in Pixel 2.

Figure 10c shows the image of fabricated TiO_2_ and Sb_2_S_3_ FROC color pixels using the thin film deposition technique. As expected, both pixels show the same color before PCM stimulus and the color of the Sb_2_S_3_ pixels is changed after annealing the sample at 250 °C. Since large spectral tunability is possible with the phase change of Sb_2_S_3_, different colors can be created by continuously annealing the FROC before the crystallization temperature of Sb_2_S_3_. The measured reflection spectrum of FROC pixels is shown in Figure 10d, where the Fano resonance of TiO_2_ and Sb_2_S_3_ (Amp) pixels matched, whereas the Fano resonance of Sb_2_S_3_ (Cry) pixel is red shifted. By using the electrical reversible color change of FROC, this method can be adapted for optical archival data storage and encrypting messages for optical steganography.

## 6 FROC as a spectrum-splitting filter

Hybrid thermal-electric power (HTEP) generation is attracting considerable interest because it offers a multifaceted approach to energy production, increasing efficiency, sustainability, and reliability while addressing environmental concerns and promoting technological innovation [50]. In HTEP, the solar spectrum can be divided into a photovoltaic (PV) band and a thermal band, where the PV band with photon energy greater than or equal to the bandgap energy ( ≥Eg ) of the PV material is directed towards a PV cell and rest of the photon energy is guided towards a solar absorber, which can be converted into thermal energy and directly used in other solar energy related applications such as water heating and sanitation. It is important to note that this energy can also be used for night-time dispatchment. However, a major issue in HTEP is the heating up of the PV cell when photons have energy <Eg and >>*Eg* incident on the PV cell. As a result, the aging rate of PV cells increases because the efficiency decreases by 0.5 % per 1 °C [51]. Another critical issue is the duck curve and curtailment problem to address the night-time dispatchment [52]. It arises from a mismatch between the solar energy peak (noon) and the demand for energy (sunset). Specifically, solar energy floods the market during the daytime and then drops off as electricity demand peaks when the sun is set. Because of this curtailment problem, grid managers sometimes turn off PV panels, which is an economic waste.

**Figure 11:**
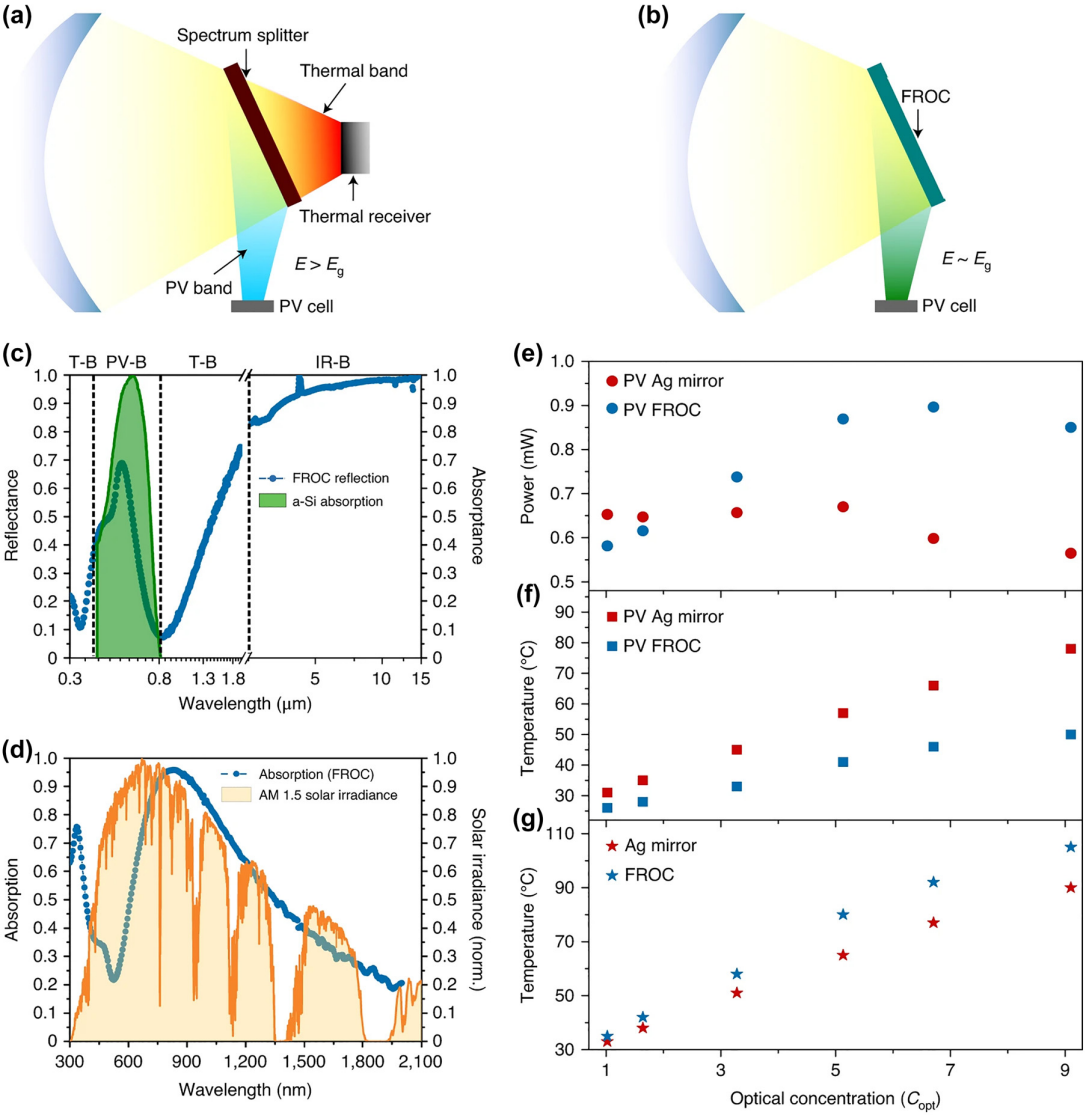
Schematic of a (a) conventional PV/solar-thermal energy conversion set-up and (b) FROC-based setup. The measured (c) reflection and (d) absorption spectra of a Ge (15 nm)–Ni (5 nm)–TiO_2_ (85 nm)–Ag (120 nm) FROC. In comparison with an Ag mirror, the measured (e) power output from a PV cell with FROC, (f) the temperature of the PV cell with FROC, and the temperature of the FROC, for different optical concentrations. Adapted with permission from ElKabbash et al. [34]. Copyright 2021, Nature Springer.

It is a challenging task to find a proper iridescence-free material system to divide the solar spectrum into the desired PV and thermal bands. In this direction, a few spectrum-splitting filters have been proposed [53]–[55]. However, these filters have several limitations: (i) they are thick (tens of micrometers), (ii) they have low emissivity and (iii) they are highly incident angle and polarization dependent. Recently, a FROC-based passive spectrum splitting filter has been proposed [34]. In a conventional PV/solar-thermal energy conversion system, the spectrum splitting filter reflects the light with photon energies >*Eg* to a PV cell and transmits the remaining energy to a separate thermal receiver (Figure 11a). However, a separate thermal absorber is not required if a properly designed FROC is used as the spectrum splitting filter, as it can reflect the solar light with energies close to the absorption band energies of silicon (a-Si) PV cells while directly absorbing the remaining photon energies (Figure 11b). That means, the FROC can effectively split the solar spectrum into a PV band (Figure 11c) and a thermal band (Figure 11d). As a result, the measured temperature of the PV cell decreased for all the optical concentrations (Figure 11f), which increases the PV cell lifetime. In addition, a PV cell operating with FROC generates more power at higher optical concentrations (Figure 11e). In short, an FROC-based spectrum splitting filter can solve the heating issue of the PV cell, and thus the power efficiency of the PV cell increases.

A dynamic spectrum splitting (DSS) filter could simultaneously solve the above-stated two issues of heating and night-time dispatchment. The operational principle of the PCM-FROC-based dynamic spectrum splitting filter is that when the PCMs in the FROC are in the Amp phase, the DSS will reflect the photon energy equivalent to the absorption spectral band of PV cell and absorb the rest of the photon energy from the solar spectrum. With structural phase switching of PCM from Amp (‘ON’ state) to Cry (‘OFF’ state), the DSS will absorb the entire photon energy from the solar spectrum. This process can be repeatedly switched electrically. By dynamically controlling the entering of light to the PV cell, it is possible to store the thermal energy in FROC during the solar energy peak. This absorbed energy can be stored as thermal energy cheaply and be dispatched at night which minimizes the economic waste. Since an ideal DSS requires perfect reflection in the ON state and complete suppression of reflection in the OFF state, these conditions can be approached by optimizing the thickness of PCM layers and controlling the loss of lossy PCM in FROC. A high-loss PCM such as Sb_2_Te_3_ could be a good choice for this purpose [56]. Using this approach, both the heating issue of the PV cells and the duck curve problem can be solved to a large extent.

## 7 Summary and outlook

The newly developed FROC and PCM-based tunable FROC were reviewed. The tunable optical properties of FROC in reflection and transmission by switching the phase of PCM layers were discussed. We conclude that the cavity layer of FROC is better to be a PCM material to achieve large Fano resonance tunability for a wide color range. The linewidth narrowing of Fano resonance to generate high-purity colors by controlling the loss of PCM was also presented. We further discussed the development of electrically tunable FROCs for dynamic color generation and optical steganography. A PCM-based FROC was proposed for the development of a dynamic spectrum splitting filter, which could mitigate the heating issue of photovoltaic cells in hybrid thermal-electric power generation. Recently developed PCM-based distributed Bragg reflectors can also be used to realize high-purity tunable structural colors through selective reflection [57]. Moreover, PCM-based tunable hyperbolic metamaterials can be used for diverse applications including biosensing and dynamic control of spontaneous emission [33], [58], [59].

Although this review has highlighted the remarkable accomplishments in tunable FROCs using chalcogenide PCMs, we foresee a continued interest in this field. The PCM-based reconfigurable FROCs with zero-static energy could play a major role in developing high-performance, video-capable, and full-color solid-state reflective displays and hybrid thermal-electric power components. In addition, tunable multiple narrow-linewidth Fano resonance of FROC can be realized using general or deterministic inverse design of layered thin film-based convolutional neural networks (CNNs) since there are large design parameter space associated with FROC [60], [61]. This FROC platform can be used to develop scalable SERS- and refractometric-based biosensors for multiplexed detection. Moreover, the wafer-scale fabrication of FROC on flexible substrate is possible due to their ultrathin scale structure [41], which can be used to develop inexpensive flexible sensors for different applications.

At the same time, critical challenges associated with PCM materials such as large area switching with fast switching speed, high endurance, and reliable multilevel switching using electrical pulse must be resolved to develop energy-efficient electrically reconfigurable FROC-based devices [11]. Realizing high endurance is the unresolved problem of PCM-based nanophotonic devices, as the endurance depends on various factors including the composition of PCM, oxidation on PCM, introduction of chemical implantation and voids during etching, inter-diffusion issues, and switching mechanism [62]. The oxidation and inter-diffusion issues can be overcome by encapsulating the PCM with a non-reactive and thermally stable dielectric material such as Si_3_N_4_, Al_2_O_3_, and ZnO. To solve the issue of inducing chemical implantation and voids during etching for nanopatterned PCM, alternate processes such as lift-off and soft etching using N_2_/CH_4_ can be used. Other strategies to achieve higher endurance are (i) selecting a PCM composition with a minimum volume shrinkage during crystallization, (ii) designing a mechanically robust structure to reduce the cyclic stress, and (iii) selecting a proper microheater design to reduce the non-uniform temperature distribution throughout the switching area. Moreover, the inter-diffusion of metal atoms into chalcogenide PCM occurs when metals such as Au, Ag, W, and Al are directly deposited on PCM, as a result, the crystallization temperature and the optical properties of PCM alter [63]. This problem in FROC can be overcome by replacing metals with extremely hard ceramics and reflecting plasmonic material such as TiN or by coating an ultra-thin and high thermal conductive dielectric spacer layer (Si_3_N_4_ or ZnO) between metal and PCM. In conclusion, further research and new disruptive design techniques are expected to realize infinitely narrow bandwidth and bound states in the continuum (BIC) like phenomena in PCM-based FROC platforms, which is still elusive on this lithography-free system.
